# Exogenous carbon inputs alleviated salt-induced oxidative stress to cotton in salinized field by improving soil aggregate structure and microbial community

**DOI:** 10.3389/fpls.2025.1522534

**Published:** 2025-03-27

**Authors:** Weidi Li, Mingtao Zhong, Haijiang Wang, Xiaoyan Shi, Jianghui Song, Jingang Wang, Wenxu Zhang

**Affiliations:** ^1^ Agricultural College, Shihezi University, Shihezi, Xinjiang, China; ^2^ Key Laboratory of Oasis Ecological Agriculture of Xinjiang Production and Construction Corps, Shihezi University, Shihezi, Xinjiang, China

**Keywords:** biochar, cotton physiology, straw, salt gradient, soil aggregates

## Abstract

High concentrations of salt ions in salinized soils not only destroy soil structure, but also inhibit crop growth. Straw and straw-derived biochar have great potential in improving soil structure, reducing soil salinity, improving soil environment, and alleviating salt stress. However, the effects and mechanisms of exogenous addition of different carbon sources on the aggregate structure and microbial community of soils with different salinization degrees in cotton fields as well as the antioxidant defense system of cotton are still unclear. In this column experiment since 15 March, 2023, three soil salt contents (1.5 (S1), 5 (S2), and 10 (S3) g/kg) and five carbon treatments (straw incorporation: 6 t/hm^2^ (C1), 12 t/hm^2^ (C2); biochar incorporation: 2.25 t/hm^2^ (B1), 4.5 t/hm^2^ (B2); CK: no straw and biochar incorporation) were designed. Then, the effects of straw and biochar incorporation on the particle size distribution of soil aggregates, bacterial and fungal communities, and cotton leaf antioxidant system in S1, S2, and S3 soils were explored. The results showed that straw and biochar incorporation, especially B2, significantly reduced the salt content of S1, S2, and S3 soils, but increased the proportion of macroaggregates by 7.01%–13.12%, 5.03%–10.24%, and 4.16%–8.31%, respectively, compared with those of CK. Straw and biochar incorporation, especially C2, increased the abundances of Actinobacteria, Acidobacteria, and Enterobacteriaceae, but decreased that of Proteobacteria, compared with CK. Besides, straw and biochar incorporation significantly increased the superoxide dismutase (SOD) and catalase (CAT) activities in salt-stressed cotton leaves, and decreased the malondialdehyde (MDA) content and peroxidase (POD) activity, compared with CK. It should be noted that the alleviating effect of straw and biochar incorporation on salt stress gradually decreased with the growth of cotton and the increase of soil salinity. In summary, straw and biochar incorporation could significantly reduce the salt content of salinized soils, increase the proportion of soil macroaggregates and microbial diversity, and alleviate the salt stress in cotton. This study will provide a scientific basis for the improvement and utilization of salinized soils.

## 1 Introduction

Soil salinization is a global environmental problem that needs to be solved urgently. About 25% of arable land worldwide is affected by soil salinization. The accumulation of large amounts of salt ions such as Na^+^ and Cl^−^ in salinized soils leads to reduced soil cohesion and stability (Xie et al., 2020), soil aggregate disintegration (Rengasamy and Olsson, 1991), and soil compaction, decreasing soil permeability (Tang et al., 2020). Soil microorganisms play an important role in soil nutrient cycling and ecosystem function (Sahu et al., 2019). However, the increase of soil salinity changes the soil microbial ecological function and metabolic pathways (Haj Amor et al., 2022), causing decreases in the ability of soil microorganisms to utilize substrates as well as the diversity and abundance of bacterial and fungal communities (Feng et al., 2024; Wei et al., 2024; Kumawat et al., 2022). Soil microbial communities have a close relationship with soil aggregates (Luo et al., 2018; Zhang et al., 2021). However, differences in the effects of different soil salinity on soil aggregate structure and microbial community and their interactions are still unclear.

Massive accumulation of base cations in the soil causes osmotic stress, ionic toxicity, and physiological drought in crops (Liu et al., 2018; Arif et al., 2020). At the same time, crops adsorb large amounts of Na^+^ from soil, and the Ca^2+^ on crop cell membrane is replaced by Na^+^ (Jin et al., 2017), causing membrane leakage and limited crops’ absorption of mineral elements. In addition, salt stress leads to the production of excessive reactive oxygen species (ROS), causing oxidative damage to crops’ cell structure and suppression on the nutrient synthesis and transport (Liu et al., 2018; Arif et al., 2020). Crops could resist salt stress through regulating its redox system, i.e., the activities of superoxide dismutase (SOD), catalase (CAT), and POD (Nefissi Ouertani et al., 2021). However, under high soil salinity conditions, the negative impacts on crops remains significant (Zhao et al., 2021).

Straw and straw-derived biochar have great potential in improving soil structure and alleviate salt stress in crops (Cen et al., 2021; Singh et al., 2021). Straw incorporation can improve soil aeration porosity, cut off soil capillaries, and prevent salt aggregation (Bai et al., 2024). In addition, straw incorporation promotes the dissolution of soluble salts in salinized soils, reducing the accumulation of salts in the soils (Ran C, et al., 2023). Biochar, with characteristics of large pores and specific surface area, can increase soil aeration and permeability, promoting salt leaching (Da Silva Mendes et al., 2021). Biochar can also adsorb salt ions on its surface or in its pores, reducing the salts in the soil (Siedt et al., 2021). The stable, loose, and porous structure and strong adsorption capacity of biochar can significantly promote the polymerization and cementation of soil particles and nutrients, promoting the formation of aggregates (Ghorbani and Amirahmadi, 2024). Duan et al. (2021) showed that Ca^2+^ and Mg^2+^ contained in biochar could replace Na^+^ in salinized soils, stimulate the combination of polyvalent cations and soil particles, increase soil aggregates, and reduce salt content. Zhao et al. (2020) reported that straw and biochar incorporation significantly increased the Shannon and Simpson diversity indexes of soil microbial communities by increasing the soil C/N ratio compared with the control. Wang et al. (2021) reported that straw incorporation significantly increased the relative abundances of Proteobacteria and Actinobacteria, while biochar incorporation significantly increased the relative abundances of Acidobacteria and Nitrospira, compared with the control. In addition, Zhu et al. (2022) found that straw and biochar incorporation significantly reduced the malondialdehyde (MDA) content and relative conductivity in crops, increased the activity of leaf antioxidant enzymes, and maintained the stability of cell membrane structure and function.

In summary, high soil salinity damages soil structure (Tang et al., 2020), inhibits soil microbial activity (Feng et al., 2024), and causes adverse impacts on plants such as metabolic disorders (Zhang W, et al., 2019). Straw and biochar incorporation can improve the physicochemical properties of salinized soils. However, it is not clear whether straw and biochar incorporation for soils with different salinization degrees can improve the physiological responses of crops to salt stress by adjusting soil aggregate structure and microbial community. Especially, the interaction effect of crops, aggregates, and microorganisms in different salinized soils mediated by straw and biochar application also needs to be further explored. In this study, straw and straw-derived biochar were incorporated into salinized soils with different salt contents, aiming to clarify their effects on the soil salinity and salt stress in cotton. The following hypotheses were proposed: (1) Straw and biochar incorporation might improve the aggregate structure and microbial community structure of soils with different salinization degrees; (2) The improved soil aggregate structure and microbial community might regulate the antioxidative system of cotton, alleviating the salt stress; (3) There might be a specific interaction between salinized soil aggregates, microorganisms, and crops mediated by straw and biochar application. This study will provide a scientific basis for the remediation and utilization of salinized soils in arid and semi-arid areas.

## 2 Materials and methods

### 2.1 Test materials

#### 2.1.1 Study area

**Figure 1:**
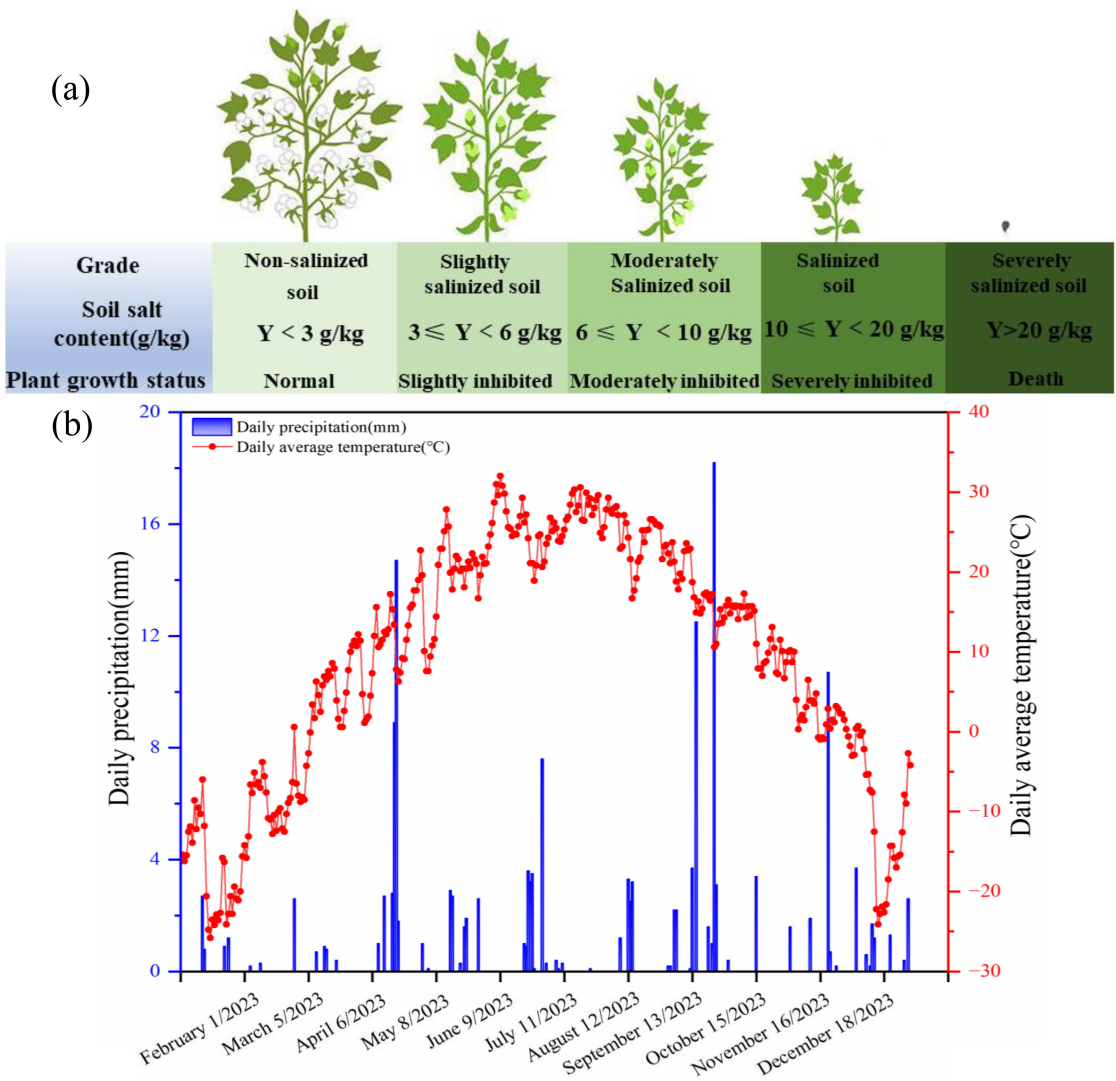
Salinized soil classifications **(a)**, precipitation and temperature **(b)** in the study area.

This study was carried out in 2023 at the experimental field of Agricultural College of Shihezi University (44°19′N, 86°58′E) in Shihezi, Xinjiang, China. The region had a temperate continental climate. The average annual precipitation was only about 220 mm, while the average annual evaporation was about 1500 mm. The average annual precipitation was only about 220 mm, while the average annual evaporation was about 1500 mm (**Figure 1**). The soil type was calcareous soil, and the texture was loam. For the soil used in this experiment, one part was collected from the nearby cotton fields under continuous cropping for more than 10 years (salt content of the 0–30 cm layer: 1.50 g·kg^−1^). The other part was collected from the salinized wasteland of Beiwucha in Manas County, Xinjiang, China (salt content of the 0–30 cm layer: 22.35 g·kg^−1^). The salt ions in the two soils were similar, mainly including Na^+^, Ca^2+^, K^+^, SO_4_^2−^, and Cl^−^. The wasteland soil and the cotton field soil were mixed in different proportions to obtain soils with different salt contents (**Table 1**).

**Table 1:** Soil physical and chemical properties.

Soil source	pH	Total salt content	Total nitrogen content	Organic matter content	Available phosphorus content	Available potassium content	Salinized soil type
		g·kg^-1^	g·kg^-1^	g·kg^-1^	mg·kg^-1^	mg·kg^-1^	
Salinized wasteland	8.86	22.35	0.43	8.46	10.53	136.79	Chloride-sulfate type
Cotton field	7.91	1.5	0.78	15.12	22.26	235.16	Chloride-sulfate type

#### 2.1.2 Biochar preparation

Cotton straw was collected from the cotton fields in the study area after the cotton harvest. After removing the roots and impurities on the surface of the straw with deionized water, the straw was air-dried, crushed, and passed through a 2 mm sieve for later use. The crushed cotton straw was pyrolyzed in a muffle furnace under oxygen limiting condition. The temperature was raised to 450 ℃ at a uniform rate for 6-hour continuous pyrolysis. After cooling to room temperature, the straw was dried at 75 ℃ for 24 h, crushed, and passed through a 2 mm sieve. Finally, the prepared cotton straw-derived biochar was collected.

### 2.2 Experimental design

**Figure 2:**
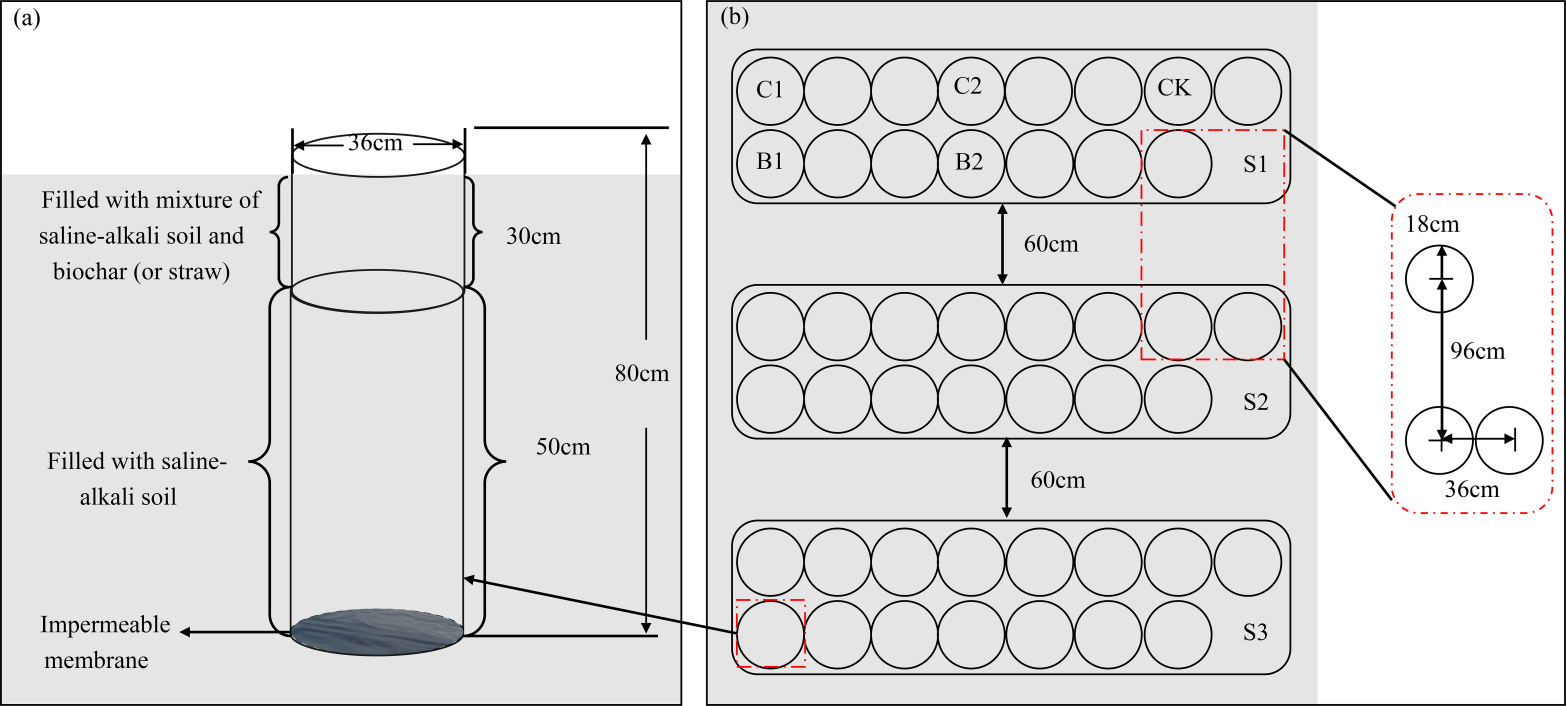
Soil column design **(a)** and layout **(b)**. S1, S2, and S3 represent soil salt content of 1.5, 5, and 10 g·kg^-1^, respectively. CK, no carbon addition; C1, 6 t·hm^-2^ of straw incorporation; C2, 12 t·hm^-2^ of straw incorporation; B1 (equal to C1), 2.25 t·hm_-2_ of biochar incorporation; B2 (equal to C2), 4.5 t·hm^-2^ of biochar incorporation. The same below.

This column experiment began on 15 March, 2023. Experimental plots were arranged in a randomized block design. By mixing the soils from the above two sources, three soil salt contents were obtained, namely non-salinization (S1): 1.5 g·kg^−1^, mild salinization (S2): 5 g·kg^−1^, and moderate salinization (S3): 10 g·kg^−1^. Cotton straw and biochar were used as exogenous carbons, and five carbon treatments were designed, including no carbon (CK), 6 t·hm^−2^ of straw incorporation (C1), 12 t·hm^−2^ of straw incorporation (C2), 2.25 t·hm^−2^ of biochar incorporation (B1, equal to C1 in carbon content), 4.5 t·hm^−2^ of biochar incorporation (B2, equal to C2 in carbon content). There were a total of 15 treatments, and each treatment had three replicates/columns. The pre-processed straw and biochar were evenly mixed into the 0–30 cm soil layer. Cotton seeds (variety Xinluzao 43) were sown after thirty days (16 April, 2023). The soil columns were 36 cm in diameter and 80 cm in height (**Figure 2**). To avoid the leaching of water and salts, the bottom of the soil columns was sealed with an impermeable membrane. One month before planting, the prepared straw and biochar were evenly mixed with the 0–30 cm soil layer. The mixed soils were layered into the soil columns while keeping the bulk density at 1.42 g·cm^−3^. Then, the columns were buried into the field, keeping the soil column top flush with the ground surface. Film mulching and drip irrigation were adopted. Four cotton were planted under one film, and each drip tape supplied water for two rows of cotton. Eight cotton seeds were evenly sown in each soil column, and four seedlings were retained in each soil column after emergence. During the whole growth period of cotton, cotton plants were irrigated every 15 days (10 times in total), and the total irrigation volume was 5400 m^3^·hm^−2^. The irrigation volume was controlled by water meters. According to the local fertilization scheme, 360 kg·hm^−2^ of urea (N, 46.2%), 105 kg·hm^−2^ of calcium superphosphate (P_2_O_5_, 46%), and 75 kg·hm^−2^ of potassium sulfate (K_2_O, 50%) were applied. Two-fifths of the fertilizers were applied before sowing, and the remaining were topdressed through the drip irrigation system after dissolving in water. Other management measures were consistent with those in the local fields.

### 2.3 Sampling

#### 2.3.1 Collection and preservation of soil samples

At the bolling stage (18 July, 2023), 200 g of soil (0–30 cm layer) was collected from each treatment by a ring cutter. After removing the soil sample part contacting with the shovel, the time, place, and amount of sampling were recorded in detail. Then, the samples were taken back to the laboratory, and shaking and tipping over were avoided when transporting samples. In the laboratory, the impurities such as roots and stones were removed from the soil samples. Then, the soil samples were placed in a ventilated place to dry naturally for the determination of soil aggregates.

In addition, soil drills were used to collect soil samples of the 0–30 cm layer (three replicates per treatment). About 100 g of soil was collected from each column. A total of 45 soil samples were collected. The soil samples were put into sealed bags, air-purged, sealed, and stored in an ice box, to prevent moisture evaporation and ensure sample activity. After taking back to the laboratory, the soil samples were divided into two parts. One part was subjected to impurity (stones and animal residues) removing and sieving (2 mm). Then, the samples were stored in sterile sealed bags for the determination of soil moisture content (SMC) and soil microbial composition. The other part was air-dried, ground, and sieved through a 2 mm sieve for the determination of soil conductivity, cation exchange capacity (CEC), and pH.

#### 2.3.2 Cotton plant sampling

In the budding (1 June, 2023), flowering (20 June), and bolling (18 July) stages of cotton, three cotton plants were randomly selected from each treatment for destructive sampling. The third and fourth leaves on the plant top were put into an ice box and brought back to the laboratory. The samples were washed separately with water, 1% hydrochloric acid, and deionized water. After drying with absorbent paper, the samples were wrapped with silver paper and stored in an ultra-low temperature refrigerator at −80°C for the determination of oxidative stress-related indicators.

### 2.4 Determination methods

#### 2.4.1 Determination of soil properties

The weight of each particle-size fraction of soil aggregates was determined by drying-sieving method. Specifically, 100 g of air-dried soil samples were sequentially sieved with sieves with pore sizes of 1, 0.5, and 0.25 mm (the sieves had a bottom and cover), to obtained the aggregates larger than 1 mm (AGG_>1_) and in the range of 1–0.5 mm (AGG_1–0.5_) and 0.5–0.25 mm (AGG_0.5–0.25_) in diameter. After sieving, the aggregates on each sieve and the soil particles with a particle size smaller than 0.25 mm (AGG_<0.25_) were separately weighed, to obtain the weight of the aggregates of different particle sizes. Then, the proportion of the aggregates of each particle size was calculated, followed by the calculation of geometric mean diameter (GMD) (Equation 1), mean weight diameter (MWD) (Equation 2), and proportion of soil aggregates greater than 0.25 mm (AGG_>0.25_) in the total aggregates (R_0.25_) (Equation 3) (Wan et al., 2024). Soil pH was determined by a pH meter (Shilu Instrument Co. Ltd, Shanghai, China) (soil: water = 1: 2.5). Soil electrical conductivity (EC) (soil: water = 1: 5) was determined by a conductivity meter (Mettler Toledo, Shanghai, China). Soil CEC was determined by ammonium acetate extraction (Bao, 2000). The SMC was determined by drying method.


(1) $$GMD=\text{exp}\left(\frac{\sum_{i=1}^{n}Wi\times \ln di}{\sum_{i=1}^{n}Wi}\right)$$

(2) $$MWD={\sum_{i=1}^{n}\bar{R}}_{i}W_{i}$$

(3) $${\text{R}}_{0.25}=\left(M_{>0.25}/M_{T}\right)\times 100\%$$

Where $\bar{R}$ _i_ is the average diameter (mm) of the aggregates in any particle size range, *Wi* is the fraction of the mass of aggregates in any particle size range to the dry mass of soil samples (%), M_>0.25_ is the weight of the aggregates greater than 0.25 mm, M_T_ is the total weight of soil samples of each treatment, *di* is the average diameter of the ith-size aggregates, *Wi* is the proportion of the mass of the ith-size aggregates in the total mass, and *n* is the number of aggregates of each size.

#### 2.4.2 Determination of antioxidant enzyme activities of cotton plant samples

The content of MDA was determined by the thiobarbituric acid method. The activity of superoxide dismutase (SOD) was determined by NBT photoreduction method. The activity of peroxidase (POD) was determined by the guaiacol method. The activity of catalase (CAT) was determine by ultraviolet absorption (Li et al., 2016).

#### 2.4.3 Determination of soil microbial composition

According to the results of a pre-test, the S1 and S3 treatments had significant differences. Therefore, the composition of soil microbial community of the two treatments were determined. The DNA extraction kit (Shanghai Meiji Biotechnology Co., Ltd., China) was used to extract microbial DNA from soil samples. The extracted DNA was used as a template, and 338F/806R and ITS1F/ITS2R were used as primers to amplify the gene sequence of soil bacterial 16S rRNA and fungal ITS. The PCR products of each sample were mixed and detected by 2% agarose gel electrophoresis. The qualified PCR library was subjected to double-end sequencing by Shanghai Meiji Biotechnology Co., Ltd. using the Illumina NovaSeq sequencing platform.

### 2.5 Statistical analysis

SPSS 26.0 (Statistical, Product and Service Solutions, Chicago, USA) was used for one-way ANOVA. Duncan’s multiple range test was used to analyze the significance of differences between means of different treatments at *p*< 0.05. Pearson correlation coefficients were calculated to analyze the correlation between indicators. Origin 2023 (Origin Lab, Massachusetts, USA) was use for charting.

## 3 Results

### 3.1 Effects of exogenous carbon addition on the physicochemical properties of different salinized soils

#### 3.1.1 Soil aggregation and stability

**Figure 3:**
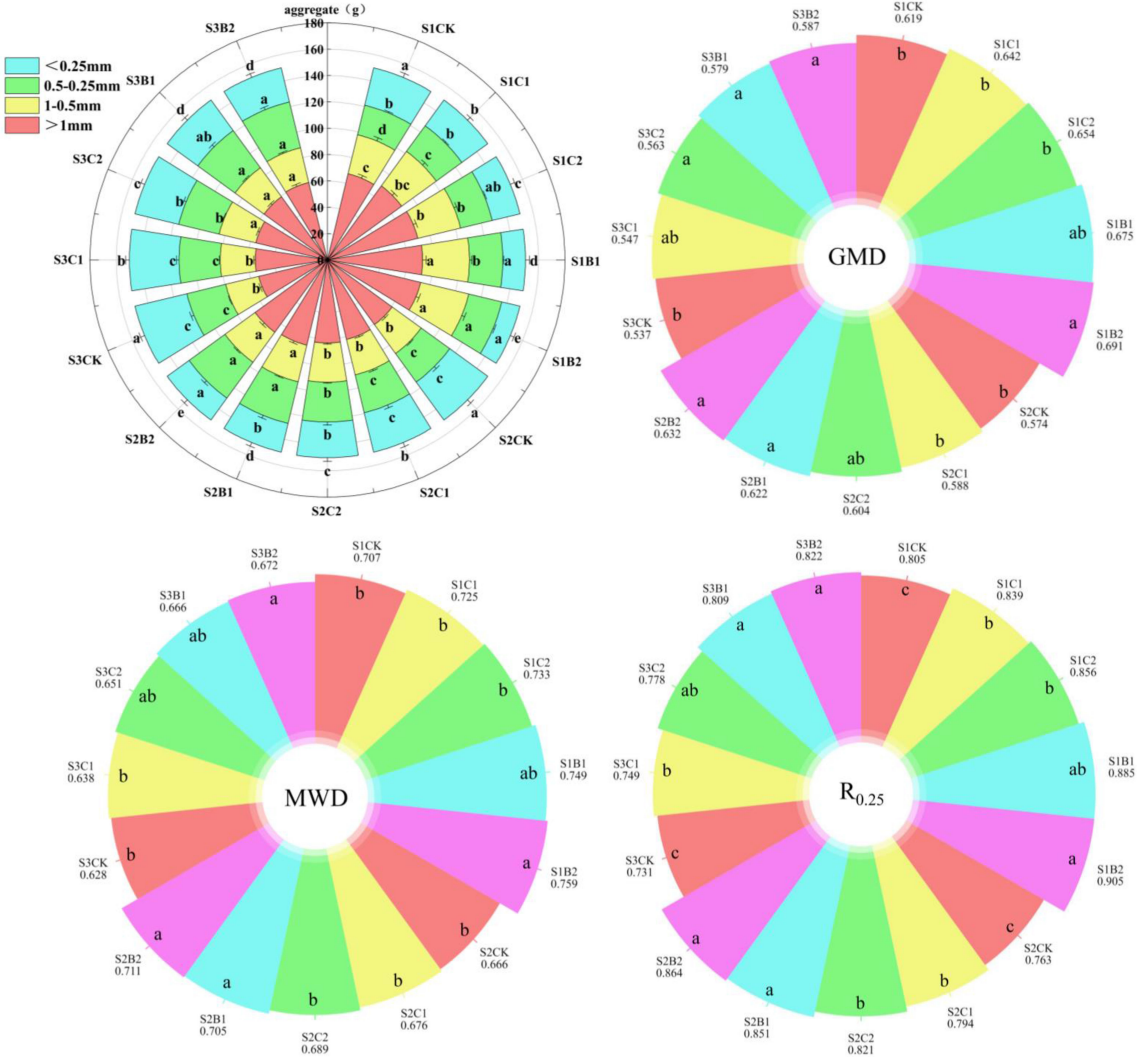
Changes of particle size distribution of aggregates in different degrees of salinized soil induced by straw and biochar incorporation. GMD, Geometric mean diameter of soil aggregates; MWD, The average weight diameter of soil aggregates R0.25: Proportion of 0.25mm particle size aggregates. Different lowercase letters in the same column indicate significant difference between groups (*p*< 0.05).

Straw and biochar incorporation had a significant effect on the number of aggregates of different particle sizes in different salinized soils (**Figure 3**). The number of AGG_>1_ and AGG_1–0.5_ decreased with the increase of soil salinity, while that of AGG_0.5–0.25_ and AGG_<0.25_ increased. Straw (C1, C2) and biochar (B1, B2) incorporation increased the number of AGG_>1_, AGG_1–0.5_, and AGG_0.5–0.25_ in the S1, S2 and S3 soils, compared with CK, and the effect of biochar incorporation was stronger than that of straw incorporation. At each soil salinity level, biochar incorporation (B1, B2) significantly increased the number of AGG_>1_, AGG_1–0.5_, and AGG_0.5–0.25_ compared with CK, with the most significant increase in the number of AGG_0.5–0.25_. Among the straw treatments, only the C2 significantly increased the number of AGG_>0.25_ compared with CK. There was no significant difference between CK and C1 treatments.

The GMD, MWD, and R_0.25_ decreased with the increase of soil salinity (**Figure 3**). The GMD, MWD, and R_0.25_ of the S2CK treatment significantly decreased by 7.75%, 5.79%, and 5.22%, respectively, and those of the S3CK treatment significantly decreased by 13.24%, 11.17%, and 9.19%, respectively, compared with those of the S1CK treatment. Straw and biochar incorporation increased the GMD, MWD, and R_0.25_ of the S1, S2, and S3 soils compared with the CK (B2 > B1 > C2 > C1 > CK). The GMD, MWD, and R0.25 of the B2 treatments (S1B2, S2B2, S3B2) significantly increased by 9.31%–11.63%, 6.75%–7.35%, and 12.42%–13.24%, respectively compared with those of the corresponding CK treatment.

#### 3.1.2 Soil moisture content, electrical conductivity, and cation exchange capacity

Straw and biochar incorporation significantly impacted soil physicochemical properties (**Table 2**). Without straw and biochar incorporation, soil EC increased significantly with the increase of soil salinity, SMC and CEC decreased significantly, and soil pH did not change significantly (**Table 2**). At each soil salinity level, soil CEC and SMC increased with the increase of straw and biochar application rate, and B2 treatments had the highest CEC and SMC, followed by C2, B1, C1, and CK. Straw and biochar incorporation significantly reduced the soil EC at each soil salinity level, and the effect of biochar incorporation was more significant (B2 > B1 > C2 > C1 > CK).

**Table 2:** Changes of soil moisture content, electrical conductivity, pH, and cation exchange capacity in different salinized soils under straw and biochar incorporation.

Index	Treatment	S1	S2	S3
Soil moisture content (%)	CK	14.81 ± 0.74e	16.04 ± 0.79d	17.3 ± 0.87bc
	C1	16.51 ± 0.82cd	16.85 ± 0.85c	17.64 ± 0.93bc
	C2	17.27 ± 0.85bc	17.92 ± 0.9b	18.37 ± 0.93ab
	B1	16.81 ± 0.85cd	17.42 ± 0.87bc	18.22 ± 0.87ab
	B2	18.47 ± 0.93ab	18.68 ± 0.95a	18.85 ± 0.94a
Electrical conductivity (mS/cm)	CK	0.85 ± 0.10i	1.61 ± 0.13e	2.52 ± 0.13a
	C1	0.56 ± 0.11j	1.41 ± 0.14f	2.37 ± 0.12b
	C2	0.51 ± 0.11j	1.32 ± 0.13f	2.28 ± 0.11c
	B1	0.42 ± 0.10j	1.24 ± 0.12g	2.10 ± 0.12d
	B2	0.29 ± 0.08k	1.15 ± 0.11h	2.05 ± 0.11d
pH	CK	7.88 ± 0.07ab	8.00 ± 0.09ab	7.93 ± 0.18ab
	C1	7.82 ± 0.13ab	7.75 ± 0.13ab	7.84 ± 0.09ab
	C2	7.72 ± 0.08ab	7.67 ± 0.16a	7.81 ± 0.13ab
	B1	7.95 ± 0.17ab	7.97 ± 0.14ab	8.02 ± 0.15ab
	B2	8.05 ± 0.11ab	8.07 ± 0.13ab	8.17 ± 0.20b
Cation exchange capacity (cmol·kg^-1^)	CK	2.3 ± 0.14f	2.23 ± 0.16f	2.22 ± 0.13f
	C1	2.63 ± 0.16e	2.83 ± 0.17de	2.53 ± 0.15e
	C2	2.99 ± 0.19cd	3.16 ± 0.21bc	2.73 ± 0.16de
	B1	2.74 ± 0.15de	2.87 ± 0.18de	2.97 ± 0.16cd
	B2	3.42 ± 0.22ab	3.56 ± 0.22a	3.25 ± 0.23ab

S1: non-salinization (1.5 g·kg^-1^), S2: mild salinization (5 g·kg^-1^), S3: moderate salinization (10 g·kg^-1^). CK: no carbon, C1: 6 t·hm^-2^ of straw incorporation, C2:12 t·hm^-2^ of straw incorporation, B1: 2.25 t·hm^-2^ of biochar incorporation (B1, equal to C1), B2:4.5 t·hm^-2^ of biochar incorporation (B2, equal to C2). All values are means ± SD (n = 3). Different lowercase letters indicate significant differences between different biochar application rates at *p*< 0.05.

### 3.2 Effects of exogenous carbon addition on antioxidant defense system in cotton

#### 3.2.1 Malondialdehyde content and peroxidase activity

**Figure 4:**
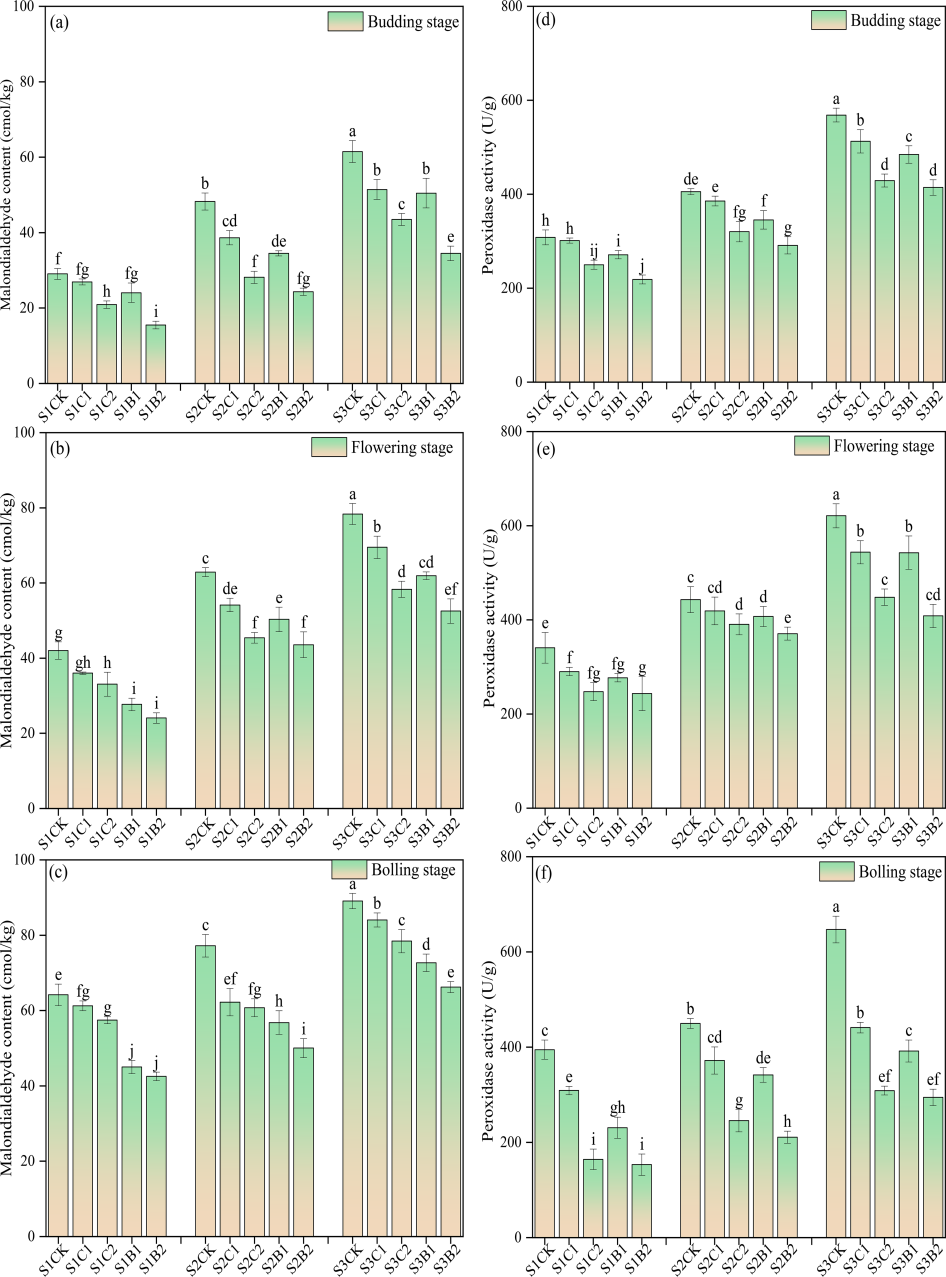
Changes of malondialdehyde (MDA) content **(a)**, budding stage; **(b)** flowering stage; **(c)** boll-forming stage) and peroxidase (POD) activity **(d)**, budding stage; **(e)** flowering stage; **(f)** boll-forming stage) with the growth of cotton in salinized soils under straw and biochar incorporation. Different lowercase letters in the same column indicate significant difference between groups (*p*< 0.05).

Under the conditions without straw and biochar incorporation, the MDA content and POD activity of cotton leaves increased with the increase of soil salinity, and showed an increasing trend with the growth of cotton, reaching the peak at the bolling stage. At each soil salinity level, the leaf MDA content of the straw and biochar treatments showed an increasing trend during the whole growth period, while the POD content increased first and then decreased. Under straw and biochar incorporation, at each soil salinity level, the MDA content and POD activity decreased with the growth of cotton. The MDA content and POD activity of the B2 treatments were significantly lower than those of the other treatments (CK > C1 > B1 > C2 > B2), and the MDA content and POD activity of the C2 treatments (S1C2, S2C2, S3C2) were significantly lower than those of CK (**Figure 4**).

#### 3.2.2 Superoxide dismutase and catalase activities

**Figure 5:**
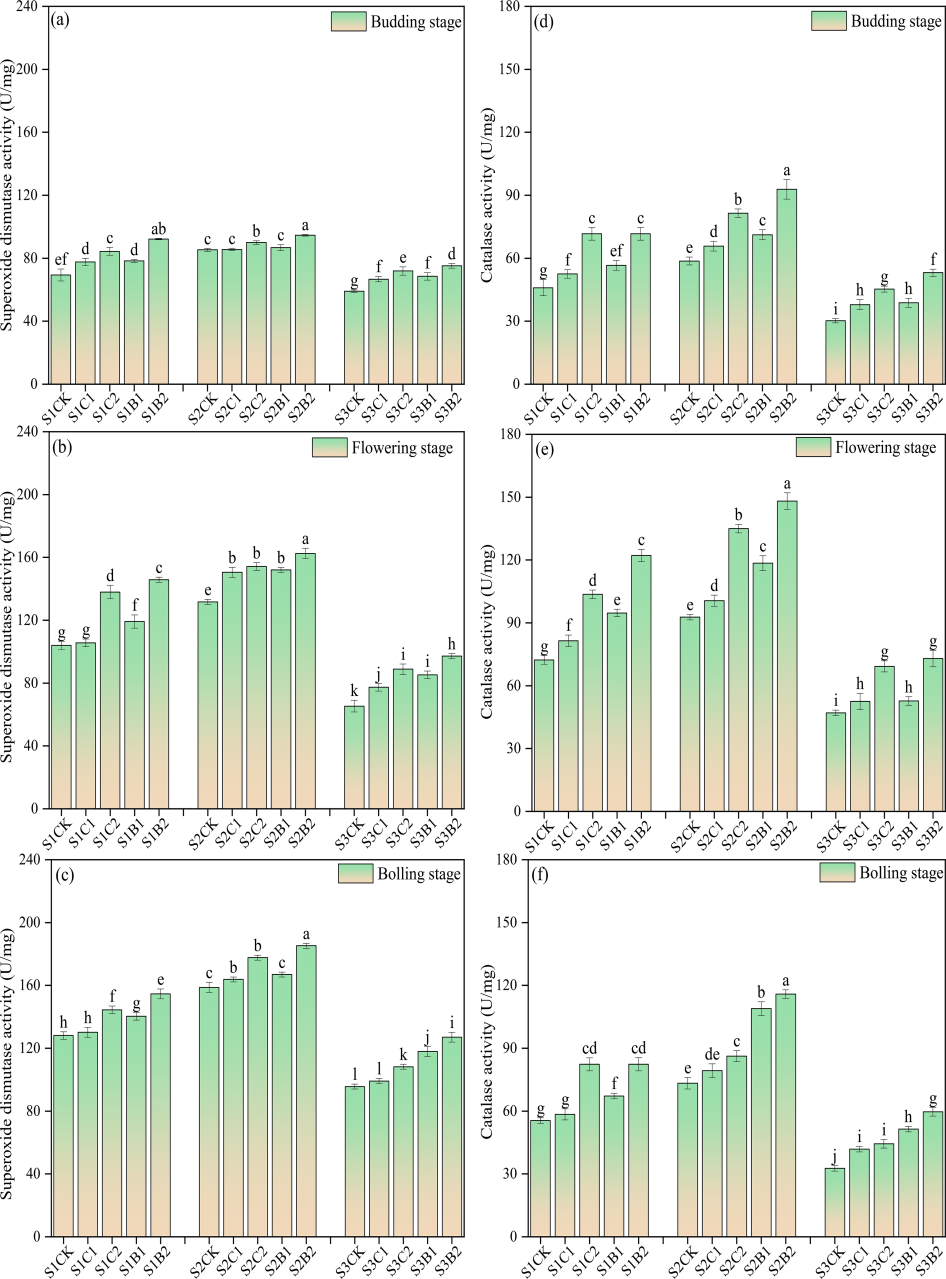
Changes of superoxide dismutase (SOD) **(a)**, budding stage; **(b)** flowering stage; **(c)** boll-forming stage) and catalase (CAT) **(d)**, budding stage; **(e)** flowering stage; **(f)** boll-forming stage) activities with the growth of cotton in salinized soils under straw and biochar incorporation. Different lowercase letters in the same column indicate significant difference between groups (*p*< 0.05).

Under the conditions without straw and biochar incorporation, the activity of SOD and CAT in cotton leaves increased with the increase of soil salinity. The SOD activity of the S1CK, S2CK, and S3CK treatments continued to increase with the growth of cotton, reaching the maximum at the bolling stage (increasing by 61.01% − 85.8% compared with that of the budding stage). However, the CAT activity of the S1CK, S2CK, and S3CK treatments showed a trend of increasing first and then decreasing, reaching the peak at the flowering stage (increasing by 56.50% − 58.62% compared with that of the budding stage). Straw and biochar incorporation increased the leaf SOD and CAT activities of cotton in salinized soils. At each salinity level, the activity of SOD and CAT increased significantly with the increase of straw and biochar application rates (CK< C1< B1< C2< B2). The activity of SOD and CAT of the B2 treatments were significantly higher than those of the other treatments, and the activity of SOD and CAT of the C2 treatments were significantly higher than those of the C1 (S1C1, S2C1, S3C1) and CK (S1CK, S2CK, S3CK) treatments. Interestingly, after the flowering stage of cotton, the activity of SOD and CAT of the S3B1 and S3B2 treatments were higher than those of S3C1 and S3C2 treatments (CK< C1< C2< B1< B2) (**Figure 5**).

### 3.3 Effects of straw and biochar incorporation on microbial activity in different salinized soils

#### 3.3.1 Changes of soil microbial diversity

**Figure 6:**
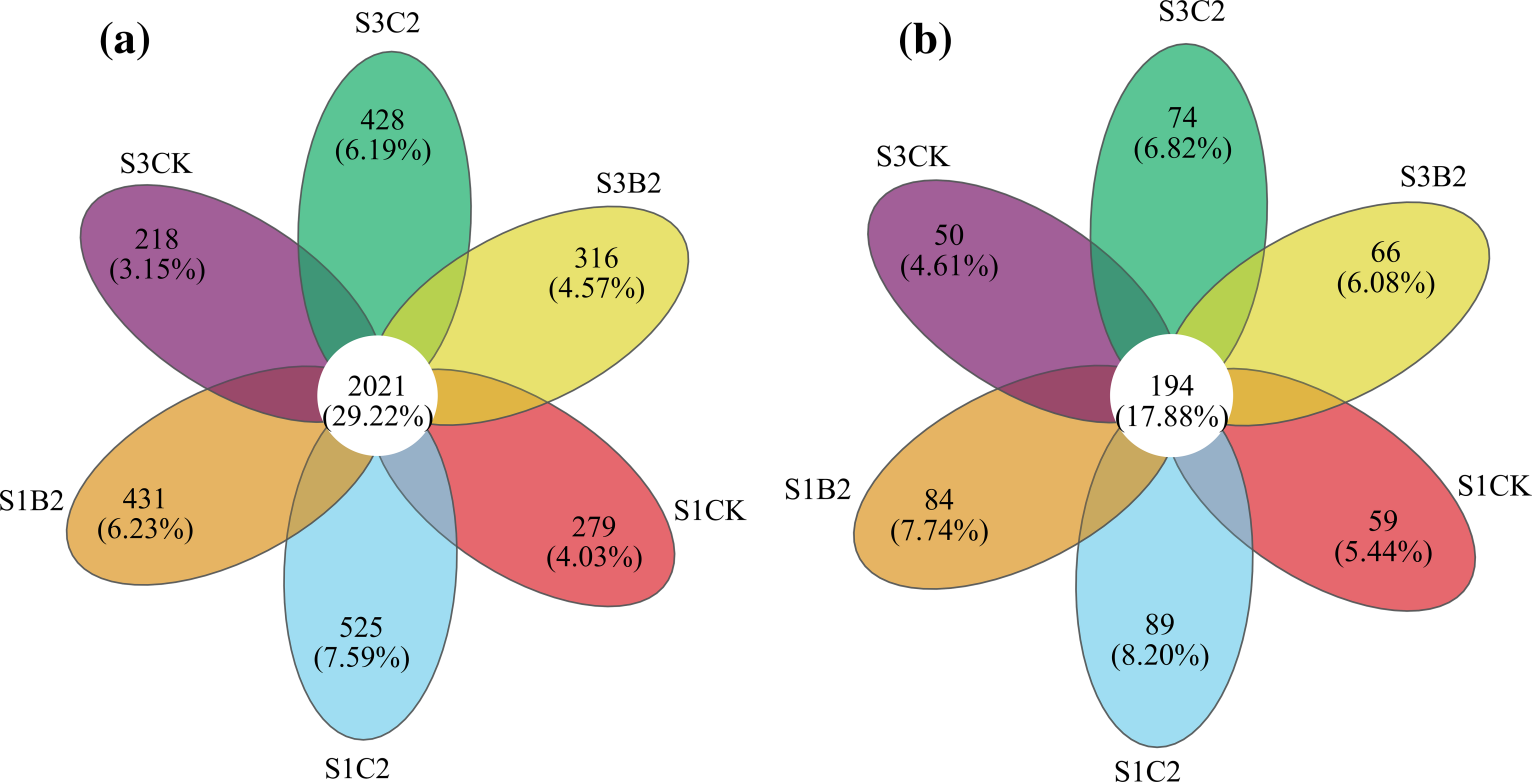
Venn diagram of bacterial **(a)** and fungal **(b)** operational taxonomic units (OTUs).

The number of bacterial Operational Taxonomic Units (OTUs) shared by all treatments was 2021, and the number of bacterial OUTs specific to S1CK, S1C2, S1B2, S3CK, S3C2, and S3B2 treatments were 279 (accounting for 12.13%), 525 (20.62%), 431 (17.58%), 218 (9.74%), 428 (17.48%), and 316 (13.52%), respectively. The number of fungal OTUs shared by all treatments was 194, and the number of fungal OTUs specific to S1CK, S1C2, S1B2, S3CK, S3C2, and S3B2 treatments were 59 (23.32%), 89 (31.45%), 84 (30.21%), 50 (20.49%), 74 (27.61%), and 66 (23.66%), respectively. The number of bacterial and fungal OUTs in each treatment decreased with the increase of soil salinity. At each soil salinity level, the effect of the straw treatments on soil bacterial and fungal communities was more significant than that of the biochar treatments (C2 > B2 > CK) (**Figure 6**).

#### 3.3.2 Changes of soil microbial community composition

**Figure 7:**
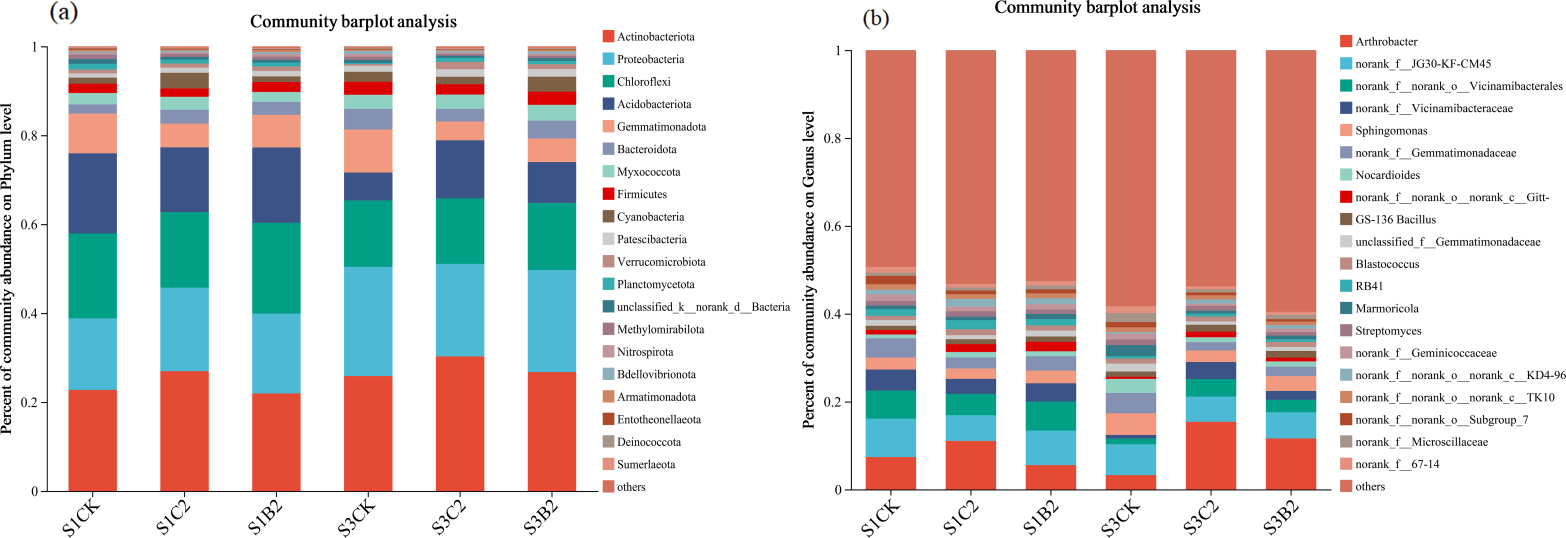
Changes of phylum-level **(a)** and genus-level **(b)** bacterial community composition in different salinized soils under straw and biochar incorporation.

At the phylum level (**Figure 7a**), the top five bacterial phyla by relative abundance in all treatments were Actinobacteria (25.74%), Proteobacteria (22.94%), Chloroflexi (15.11%), Acidobacteria (9.18%), and Gemmatimonadetes (5.31%), accounting for 78.28% in total. The relative abundances of Proteobacteria and Bacteroidete of the S1CK, S2CK, and S3CK treatments increased with the increase of soil salinity, while those of Chloroflexi and Acidobacteria showed an opposite variation trend. At each soil salinity level, the relative abundances of Actinobacteria and Acidobacteria of the C2 (S1C2, S2C2, S3C2) and B2 (S1B2, S2B2, and S3B2) treatments increased compared with those of the CK treatments, while the relative abundances of Gemmatimonadetes and Bacteroidetes decreased. Besides, there was no significant change in the relative abundances of Proteobacteria and Chloroflexi. In general, the effect of the C2 treatments on the relative abundances of soil bacteria was stronger than that of the B2 treatments.

At the genus level (**Figure 7b**), the top five dominant bacterial genera by relative abundance were *Arthrobacter* (11.61%), *JG30-KF-CM45* (5.95%), *Vicinamibacterales* (3.38%), *Vicinamibacteraceae* (2.86%), and *Sphingomonas* (1.98%), accounting for 25.33% in total. The relative abundances of *Arthrobacter*, *JG30-KF-CM45*, *Aicinamibacterales*, and *Vicinamibacteraceae* of the S1CK, S2CK, and S3CK treatments reduced with the increase of soil salinity, while that of *Sphingomonas* increased. The relative abundance of *Arthrobacter* of the C2 (S1C2, S3C2) and B2 (S1B2, S3B2) treatments increased (S1C2 > S1B2; S3C2 > S3B2) compared with that of the S1CK treatment. The relative abundance of *Vicinamibacterales* of the S3C2 and S3B2 treatments increased, while that of *Sphingomonas* decreased, compared with those of the S3CK treatment. There was no significant change in the relative abundance of *JG30-KF-CM45*.

**Figure 8:**
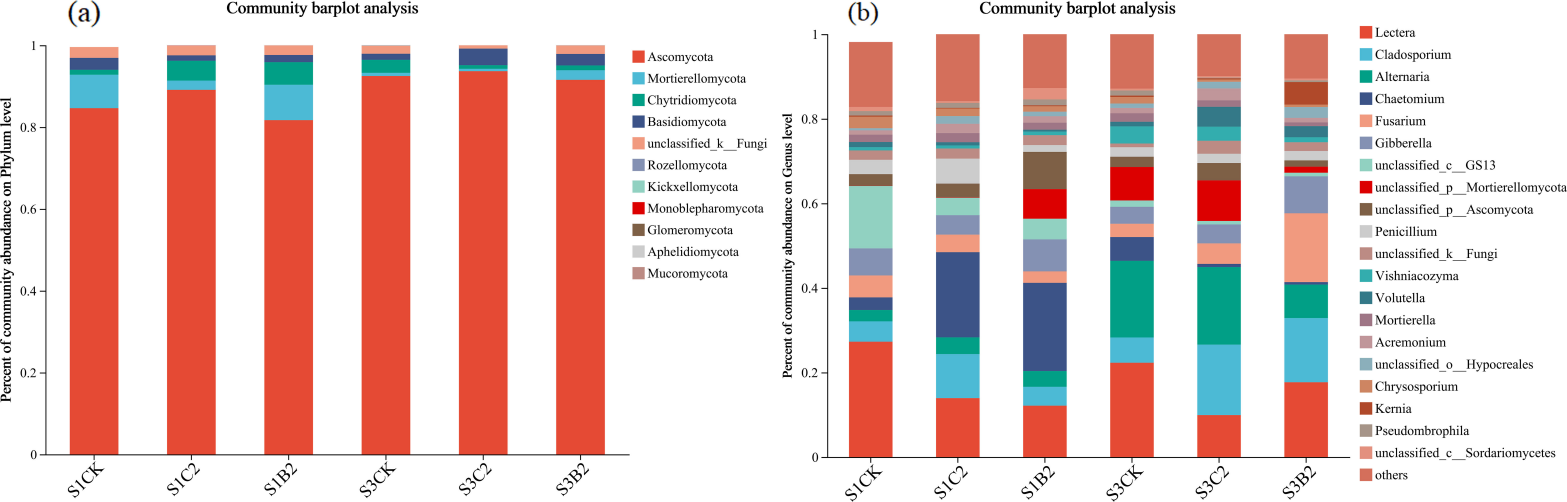
Changes of phylum-level **(a)** and genus-level **(b)** fungal community composition in different salinized soils under straw and biochar incorporation.

At the phylum level (**Figure 8a**), the top five fungal phyla by relative abundance in all treatments were Ascomycota (89.75%), Mortierella (7.23%), Chytridiomycota (1.33%), Basidiomycota (1.02%), and unclassified_k_Fungi (0.55%), accounting for about 90% in total. The relative abundance of Ascomycota of the S1CK, S2CK, and S3CK treatments increased with the increase of soil salinity, while that of Ascomycota, Chytridiomycota, Basidiomycota, and unclassified_k_Fungi decreased. The relative abundances of Ascomycota and Chytridiomycota of the S1C2 treatment and Mortierella and Chytridiomycota of the S1B2 treatment increased, while the relative abundance of Basidiomycetes of the S1C2 treatment decreased, compared with those of the S1CK treatment. The relative abundance of Basidiomycetes of the S3C2 and S3B2 treatments increased compared with that of the S3CK treatment, while that of Ascomycota did not change significantly.

At the genus level (**Figure 8b**), the top five dominant genera by relative abundance were *Lectera* (15.68%), *Cladosporium* (12.18%), *Alternaria* (10.81%), *Chactomium* (9.36%) and *Fusarium* (8.15%), accounting for 56.24% in total. The relative abundances of *Lectera* and *Fusarium* of the S1CK, S2CK, and S3CK treatments reduced with the increase of soil salinity, while those of *Alternaria* and *Chactomium* increased. The relative abundances of *Cladosporium* and *Chactomium* of the S1C2 and S1B2 treatments increased (S1C2 > S1B2) compared with those of the S1CK treatment, while the relative abundance of *Lectera* decreased. The relative abundances of *Cladosporium* and *Fusarium* of the S3C2 and S3B2 treatments increased (S3B2 > S3C2), while the relative abundance of *Lecter*a decreased, compared with those of the S3CK treatment. The relative abundance of *Alternaria* of the S3B2 treatment reduced compared with that of the S3CK treatment, while there was no significant difference between S3C2 treatment and S3CK treatment.

#### 3.3.3 Microbial species differences

**Figure 9:**
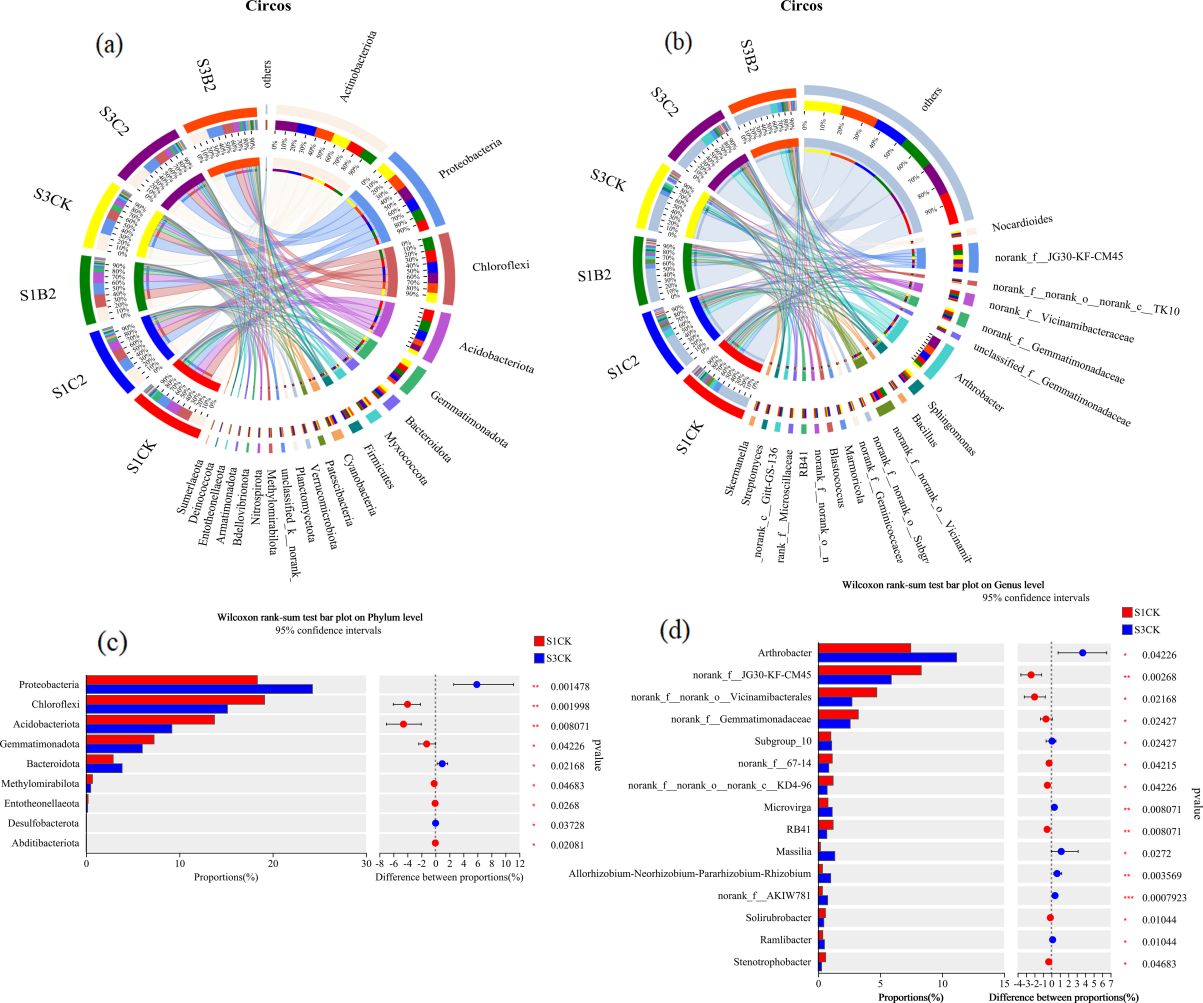
Analysis of differences in bacterial abundance in different salinized soils under straw and biochar incorporation. **(a)** Differences in the relative abundances of bacterial phyla under straw and biochar incorporation; **(b)** Differences in the relative abundances of bacterial genera under straw and biochar incorporation; **(c)** Differences in the relative abundances of bacterial phyla under salt treatments; **(d)** Differences in the relative abundances of bacterial genera under salt treatments.

At the phylum level (**Figure 9a**), the relative abundances of Actinobacteria (19%), Chloroflexi (20%), and Acidobacteria (24%) were significantly higher in the S1C2 treatment than in other treatments (S1C2 > S3C2 > S1B2 > S1CK > S3B2 > S3CK). However, the relative abundances of Proteobacteria (20%) and Blastomonas (23%) were significantly higher in the S3CK treatment than in other treatments. At the genus level (**Figure 9b**), the relative abundances of *Arthrobacter* (20%) and *Vicinamibacteraceae* (27%) were significantly higher in the S1C2 treatment than in other treatments. The relative abundances of *Vicinamibacterales* (26%) and *JG30-KF-CM45* (21%) were significantly higher in the S1CK treatment than in other treatments. The relative abundance of *Sphingomonas* (26%) was significantly higher in the S3CK treatment than in other treatments. In general, straw incorporation treatments had a more significant effect on soil bacterial community structure than biochar incorporation treatments.

At the phylum level (**Figure 9c**), the relative abundances of Proteobacteria (*p* = 0.0014) and Bacteroidetes (*p* = 0.0268) of the S3 treatments (S3C1, S3C2, S3B1, S3B2) were significantly higher than those of the S1 treatments (S1C1, S1C2, S1B1, S1B2), while the relative abundances of Chloroflexi (*p* = 0.0019), Acidobacteria (*p* = 0.008), and Gemmatimonadetes (*p* = 0.0422) were significantly lower than those of the S1 treatments. At the genus level (**Figure 9d**), the relative abundances of *Arthrobacter* (*p* = 0.0422), *Subgroup_10* (*p* = 0.0242), *Microvirga* (*p* = 0.0080), *Massilia* (*p* = 0.0272), *AKIW781* (*p* = 0.0007), and *Ramlibacte*r (*p* = 0.0104) of the S3 treatments were significantly higher than those of the S1 treatments. However, the relative abundances of *G30-KF-CM45* (*p* = 0.0026), *Vicinamibacterales* (*p* = 0.0216), *Gemmatimonadaceae* (*p* = 0.0242), *KD4-96* (*p* = 0.0422), *RB41* (*p* = 0.0080), *Soilrubrobacter* (*p* = 0.0104), and *Stenotrophobacter* (*p* = 0.0468) of the S3 treatments were significantly lower than those of the S1 treatments.

**Figure 10:**
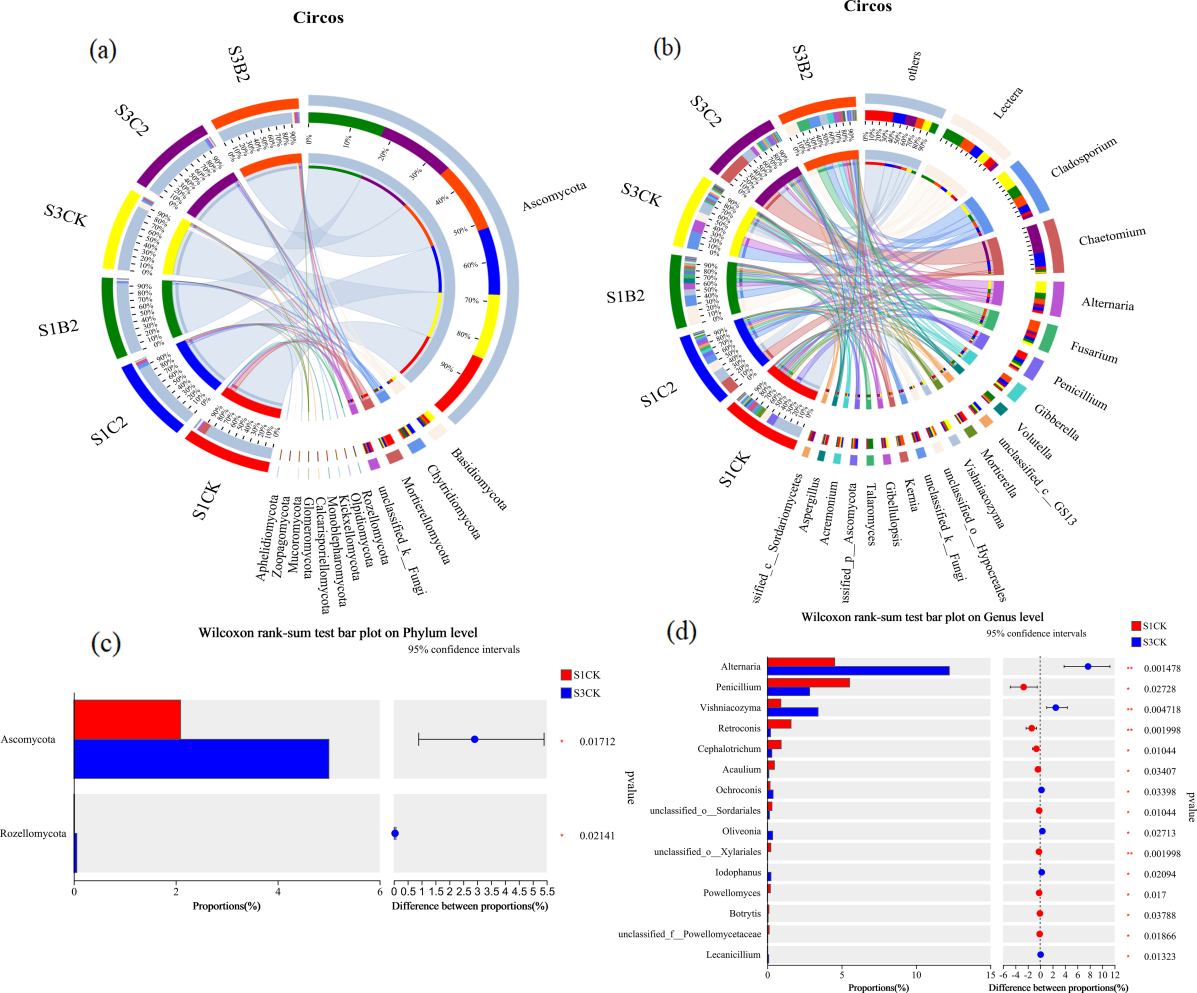
Analysis of differences in fungal abundance in different salinized soils under straw and biochar incorporation. **(a)** Differences in the relative abundances of fungal phyla under straw and biochar incorporation; **(b)** Differences in the relative abundances of fungal genera under straw and biochar incorporation; **(c)** Differences in the relative abundances of fungal phyla under salt treatments; **(d)** Differences in the relative abundances of fungal genera under salt treatments.

At the phylum level (**Figure 10a**), the relative abundances of Mortierellomycota (31%), unclassified_k_Fungi (29%), and Basidiomycota (29%) were significantly higher in the S1C2 treatment than in other treatments. The relative abundance of Chytridiomycota (20%) was significantly higher in the S3C2 treatment than in other treatments. The relative abundance of Ascomycetes (19%) was significantly higher in the S3CK treatment than in other treatments. At the genus level (**Figure 10b**), the relative abundance of *Lectera* (32%) was significantly higher in the S1C2 treatment than in other treatments. The relative abundance of *Cladosporium* (34%) was significantly higher in the S3CK treatment than in other treatments. The relative abundances of *Alternaria* (36%), *Chactomium* (40%), and *Fusarium* (24%) was significantly higher in the S3C2 treatment than in other treatments. In general, the effect of straw incorporation treatment on soil fungal community structure was more significant than that of biochar incorporation treatment.

At the phylum level (**Figure 10c**), there were significant differences in the relative abundances of Ascomycota and Rhodomycota between S1, S2, and S3 treatments, and the relative abundances of Ascomycota (*p* = 0.0017) and Rhodomycota (*p* = 0.0214) of the S3 treatment were significantly higher than those of the S1 treatments. At the genus level (**Figure 10d**), the relative abundances of *Penicillium* (*p* = 0.0014), *Vishniacozyma* (*p* = 0.0047), and *Ochroconis* (*p* = 0.0339) of the S3 treatments were significantly higher than those of the S1 treatments, while the relative abundances of *Alternaria* (*p* = 0.0272), *Retroconis* (*p* = 0.0019), *Cephalotrichum* (*p* = 0.0104), *Acaulium* (*p* = 0.0340), and *Sordariales* (*p* = 0.0104) were significantly lower than those of the S1 treatments.

**Figure 11:**
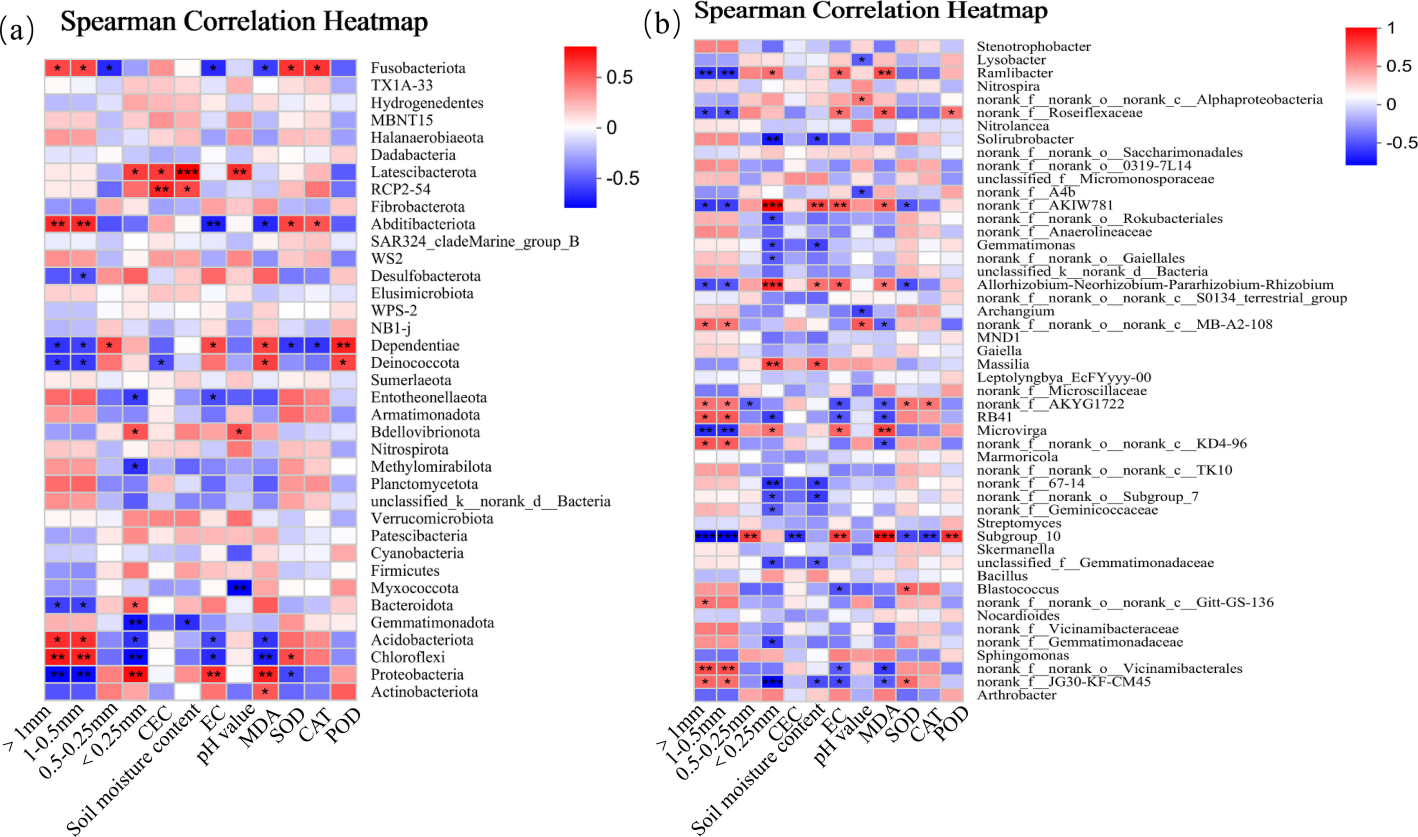
Correlation analysis of soil bacterial community (**(a)** bacterial phyla; **(b)** bacterial genera) and environmental factors.

At the phylum level (**Figure 11a**), there were significant correlations between dominant soil bacterial phyla (i.e., Acidobacteria, Abditibacteriot, Strangeococcus, Actinobacteria, Chloroflexi, and Proteobacteria) and environmental factors (soil factors and cotton parameters). Specifically, the relative abundance of Acidobacteria was positively correlated with AGG_>1_ number, AGG_1−0.5_ number, leaf SOD activity, and leaf CAT activity (*p<* 0.05), and negatively correlated with AGG_<0.25_ number, soil EC, and leaf MDA content (*p<* 0.05). The relative abundance of Abditibacteriot was positively correlated with the number of AGG_>1_ and AGG_1−0.5_ (*p<* 0.01), and negatively correlated with soil EC (*p<* 0.01) and leaf MDA content (*p<* 0.05). The relative abundance of Strangeococcus was negatively correlated with soil EC (*p<* 0.05), and positively correlated with the number of AGG_>1_ and AGG_1−0.5_, leaf SOD activity, and CAT activity. The relative abundances of Actinobacteria and Chloroflexi were positively correlated with the number of AGG_>1_ and AGG_1−0.5_ (*p<* 0.05), and negatively correlated with AGG_<0.25_ number, soil EC, and leaf MDA content (*p<* 0.05). The relative abundance of Proteobacteria was negatively correlated with the number of AGG_>1_ and AGG_1−0.5_ (*p<* 0.01), and positively correlated with soil EC and leaf MDA content (*p<* 0.01). At the genus level (**Figure 11b**), the relative abundances of dominant bacterial genera *Ramlibacter*, *Roseiflexaceae*, *AKIW781*, *AKYG1722*, *RB41*, *Microvinga*, *Subgroup_10*, *Blastococcus*, *Vicinamibacterales*, and *JG30-KF-CM45* were significantly correlated with environmental factors. Among them, the relative abundance of *AKIW781* was positively correlated with the number of AGG_<0.25_ (*p<* 0.001). The relative abundance of *Microvinga* was negatively correlated with the number of AGG_>1_ and AGG_1−0.5_, and positively correlated with leaf MDA content. The relative abundance of *Ubgroup_10* was negatively correlated with the number of AGG_>1_ and AGG_1−0.5_ (*p<* 0.01), and positively correlated with leaf MDA content (*p<* 0.001). The relative abundance of *Vicinamibacterales* was positively correlated with the number of AGG_>1_ and AGG_1−0.5_. The relative abundance of *JG30-KF-CM45* was positively correlated with the number of AGG_>1_ and AGG_1−0.5_ (*p<* 0.05), and negatively correlated with the number of AGG_<0.25_ (*p<* 0.001).

**Figure 12:**
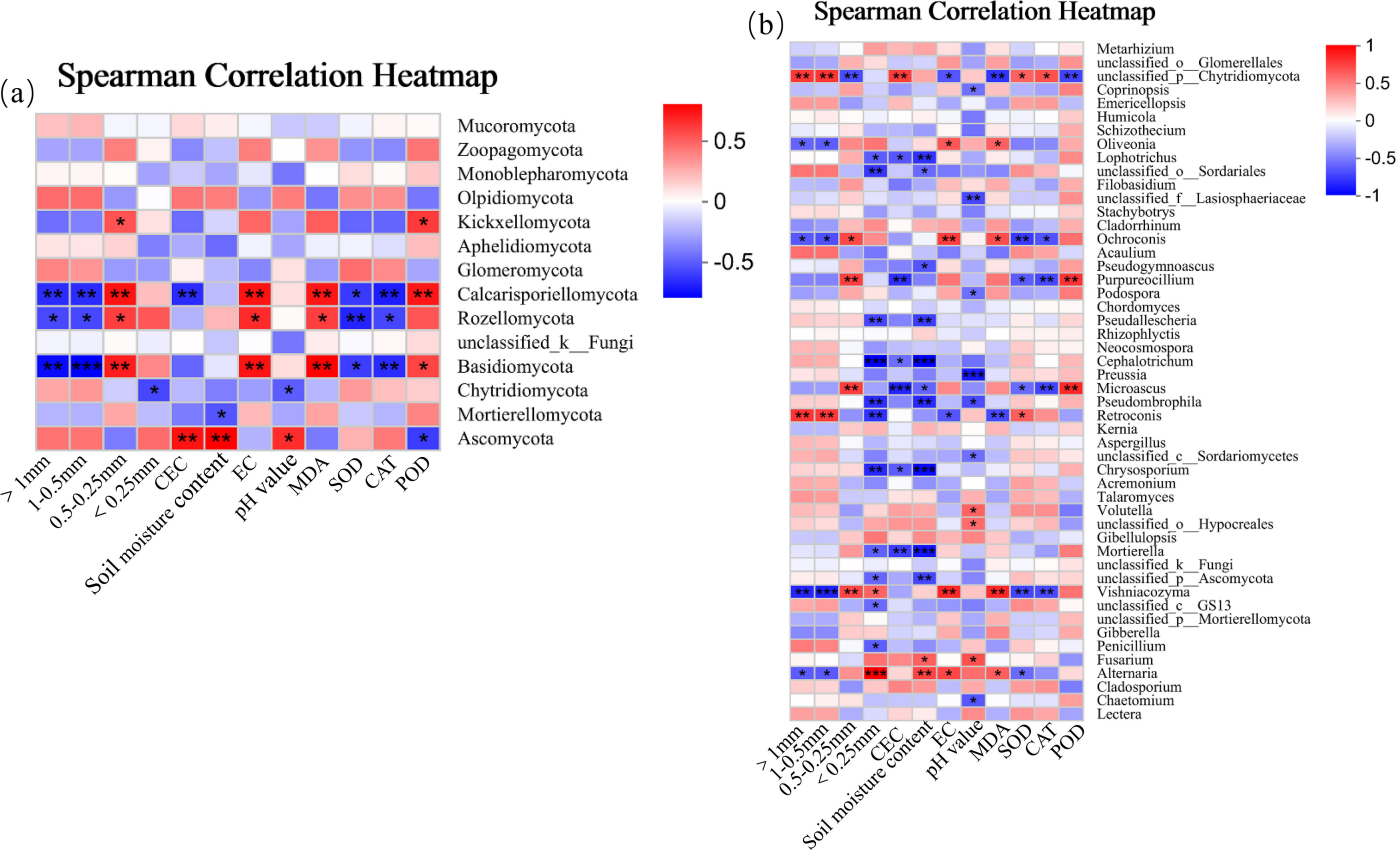
Correlation analysis of soil fungal community (**(a)** fungal phyla; **(b)** fungal genera) and environmental factors.

At the phylum level (**Figure 12a**), there were significant correlations between dominant soil fungi (i.e, Ascomycota, Calcosporangia, and Basidiomycetes) and environmental factors. Among them, the relative abundances of Ascomycota and Calcosporangia were negatively correlated with AGG_>1_ number, AGG_1−0.5_ number and leaf CAT activity (*p<* 0.01), and positively correlated with AGG_0.5−0.25_ number, soil EC, and leaf POD activity (*p<* 0.01). The relative abundance of Roselle was negatively correlated with AGG_>1_ number, AGG_1−0.5_ number and leaf CAT activity (*p<* 0.05), and positively correlated with AGG_0.5−0.25_ number, soil EC, and leaf POD activity (*p<* 0.05). The relative abundance of Basidiomycota was positively correlated with CEC and SMC (*p<* 0.01), and negatively correlated with leaf POD activity (*p<* 0.05).

At the genus level (**Figure 12b**), there were significant correlations between dominant soil fungal genera (i.e., *Chytridiomycota*, *Ochroconis*, *Cladosporium*, *Purpureoclilium*, *Microascus*, *Vishniacozyma*, *Retroconis*, and *Alternaria*) and environmental factors. Among them, the relative abundances of *Chytridiomycota* and *Retroconis* were positively correlated with AGG_>1_ number, AGG_1−0.5_ number and CEC (*p<* 0.01), and negatively correlated with leaf MDA content (*p<* 0.01). The relative abundance of *Chytridiomycota* was negatively correlated with the number of AGG_0.5−0.25_ (*p<* 0.01). The relative abundance of *Retroconis* was negatively correlated with the number of AGG_<0.25_(*p<* 0.05). The relative abundance of *Ochroconis* was positively correlated with soil EC (*p<* 0.01), AGG_0.5−0.25_ number (*p<* 0.05), and leaf MDA content (*p<* 0.05), and negatively correlated with leaf SOD activity (*p<* 0.01). The relative abundances of *Purpureoclilium* and *Microascus* were positively correlated with AGG_0.5−0.25_ number and leaf POD activity (*p<* 0.01), and negatively correlated with CEC and CAT activity (*p<* 0.01). The relative abundance of *Vishniacozyma* was negatively correlated with AGG_>1_ number, AGG_1−0.5_ number, leaf SOD activity, and CAT activity (*p<* 0.01), and positively correlated with AGG_0.5−0.25_ number, soil EC, and leaf MDA content (*p<* 0.01).

## 4 Discussion

### 4.1 Effects of straw and biochar incorporation on the physicochemical properties of different salinized soils

Soil salinization greatly affects the productivity of cultivated lands. High soil salinity damages soil aggregate structure, leading to soil compaction and reduced permeability (Wong et al., 2010). This may ultimately lead to the abandonment of cultivated lands. Previous studies have shown that straw and biochar incorporation can significantly reduce soil salinity, improve soil environment, and promote the leaching of base ions in salinized soils (Yue et al., 2016; Xie et al., 2017). Similarly, this study found that straw and biochar incorporation significantly reduced soil EC, but increased soil CEC (**Table 2**). This may be due the fact that (1) straw and biochar incorporation increase soil porosity and aeration (Artiola et al., 2012) and accelerate the leaching of Na^+^. (2) Biochar has a large surface area and strong adsorption capacity, thus it can adsorb base ions and reduce salt content in salinized soils (Yang et al., 2024). (3) Straw and biochar have a large number of oxygen-containing functional groups such as hydroxyl and carboxyl groups, which can combined with Ca^2+^, Mg^2+^, K^+^, and Al^3+^ to stabilize in the soil. This may be the main reason for the increase of CEC. This study also found that straw incorporation reduced soil pH compared with CK, while biochar incorporation had no significant impact on soil pH. This may be due to that the soil pH (8.35) in the study area is high causing an insignificant effect of biochar incorporation on soil pH.

Soil aggregate is not only a fundamental unit of soil structure, but also an important carrier of soil moisture, organic matter, and nutrients. Its stability and the proportion of each size of particles largely determine the soil structure, water and fertilizer retention capacity (Guidi et al., 2017). In this study, the number of soil macroaggregates in high-salinity soil was significantly lower than that in low- and medium-salinity soils, and the GMD, MWD, and R_0.25_ were smaller. This is consistent with the results of Duan et al. (2021). This may be due to the fact that the Na^+^ content of high-salinity soil is higher than that of low- and medium-salinity soils, and the larger hydration radius of Na^+^ weakens the force between soil particles, thus reducing the number of macroaggregates in the soil (Shen et al., 2024). In addition, the large accumulation of Na^+^ reduces the soil osmotic potential, resulting in loose aggregate structure and soil compaction (Guo et al., 2019). Straw and biochar incorporation can increase the number and average diameter of macroaggregates and the stability of soil aggregates by reducing the soil Na^+^ content and increasing organic and inorganic cementing substances (Zheng et al., 2018). In this study, straw and biochar incorporation increased the number of soil macroaggregates in low-, medium-, and high-salinity soils by 7.01%−13.12%, 5.03%−10.24%, and 4.16%−8.31%, respectively, compared with CK (**Figure 3**). Among them, the increase in the soil macroaggregates in low-salinity soil was the largest, and the effect of biochar incorporation was better than that of straw incorporation. However, the mechanisms of action of straw and biochar incorporation are different. Lignin and protein released during straw decomposition are important factors to maintain the stability of aggregates (Xie et al., 2020). The surface of biochar is rich in divalent cations such as Ca^2+^ and Mg^2+^, which can replace excess Na^+^ in salinized soil and inhibit the Na^+^-induced cohesion reduction of soil aggregates (Duan et al., 2021). In addition, biochar can also act as an intermediate between soil organic matter and soil minerals, promoting the formation of an organic matter-biochar-mineral complex through adsorption and cationic bridging (Kim et al., 2016). This ultimately enhances the stability of aggregates.

### 4.2 Response of antioxidant defense system of cotton to salt stress and exogenous carbon addition

Soil salinization causes salt stress, which limits crop growth by inducing physiological drought, ion toxicity, and oxidative damage (Bose et al., 2014; Nefissi Ouertani et al., 2021). Kesawat et al. (2023) found that salt stress led to excess accumulation of reactive oxygen species (ROS) in crop organelles (i.e., mitochondria and peroxisomes), and disrupted cell membrane integrity and functional molecules such as nucleic acids, proteins, and lipids. In this study, the MDA content in cotton leaves continued to increase under high salinity treatment. This is due to the fact that under salt stress, the reaction of excess ROS with unsaturated fatty acids on the cell membrane causes increasing membrane lipid peroxidation in cotton leaves (Zhao et al., 2023). In addition, excess Na^+^ competes with K^+^ and Ca^2+^ to destroy the ion balance in crops, leading to a large accumulation of MDA (Hussain et al., 2021). In this study, the activities of POD, SOD, and CAT in cotton of the straw and biochar incorporation treatments significantly increased compared with those of the CK treatment. This indicates that crops can resist salt stress by regulating the antioxidant defense system to remove excess ROS and reduce the damage of oxidative stress to cells (Hossain and Dietz, 2016).

In this study, the straw and biochar incorporation significantly increased the leaf SOD and CAT activities in cotton in salinized soils (**Figure 5**), and decreased the MDA content and POD activity, compared with CK (**Figure 4**). This is consistent with the results of previous studies (Abbas et al., 2022; Hamoud et al., 2022; Zhang J, et al., 2019). This may be due to the fact that straw and biochar contains rich nutrients, which can promote the nutrient absorption of cotton roots and provide a material basis for the synthesis of SOD and CAT. SOD catalyzes superoxide anions to undergo dismutation reaction and decomposes superoxide anions (O_2_^−^) into H_2_O_2_ and water, and CAT further decomposes H_2_O_2_ into H_2_O and (O_2_). The two synergistically alleviate the oxidative stress caused by excess ROS under salt stress (Hossain and Dietz, 2016; Muchate et al., 2016). In addition, straw and biochar incorporation can promote the leaching of soil base ions (especially Na^+^) by improving soil physical properties (Huang et al., 2023; Jin et al., 2024), reduce the absorption of Na^+^ by cotton, thus alleviating the inhibition of soil salts on the synthesis of SOD and CAT. It should be noted that in this study, the leaf SOD and POD activities continued to increase with the growth of cotton, while the CAT activity increased first and then decreased (**Figures 4d-f** and **5**). This may be due to the continuous accumulation of ROS in cotton caused by salt stress. Excess Na^+^ intake in cotton affects the molecular structure and active site of CAT, resulting in a decrease in CAT activity (Mishra et al., 2013).

### 4.3 Effects of straw and biochar incorporation on microbial community in different salinized soils

Soil salts greatly impact the composition and diversity of soil microbial communities (Rath and Rousk, 2015). Straw and biochar incorporation can regulate the microbial community structure by reducing soil salinity and increasing soil fertility. This study found that the relative abundances of Proteobacteria (*p* = 0.0014) and Bacteroidetes (*p* = 0.0268) increased with the increase of soil salinity, while those of Chloroflexi (*p* = 0.0019), Acidobacteria (*p* = 0.008), and Gemmatimonadetes (*p* = 0.0422) decreased (**Figure 9c**). This may be due to the fact that Proteobacteria can absorb and accumulate compatible solutes such as glycine betaine, and maintain normal metabolic activity under salt stress by changing osmotic balance and antioxidant defense system. Bacteroidetes is an aerobic organism, which can cooperate with other bacteria to increase nutrient uptake by using the matrix in salinized soil (Li et al., 2021) to resist salt stress. Besides, soil microbial competition is also one of the important reasons. High soil salinity inhibits the activity of non-salt-tolerant microorganisms, thus enabling salt-tolerant microorganisms (such as Proteobacteria and Bacteroidetes) to obtain greater living space and more resources (Zheng et al., 2017).

Actinobacteria and Ascomycota, as the main bacterial and fungal phyla, play a very important role in carbon and nitrogen cycling in soil ecosystems (Chen et al., 2023; Xu et al., 2012). Acidobacteria and Chytridiomycota can decompose organic matters such as cellulose and chitin (Ran T, et al., 2023). Mortierella significantly affects the contents of available nitrogen and available phosphorus in soil. Song et al. (2017) and Guan et al. (2022) showed that the total carbon content, total nitrogen content, and C/N ratio in the soil under straw and biochar incorporation significantly increased by 31.3%, 40%, and 44%, respectively compared with the control. In this study, straw and biochar incorporation increased the relative abundances of beneficial bacterial (i.e., Actinobacteria and Acidobacteria) and fungal (i.e., Enterobacteriaceae) phyla in salinized soils (**Figures 6**-**8**). This may be due to that straw and biochar incorporation regulate carbon and nitrogen cycles and C/N ratio in salinized soils (Dong et al., 2023). Importantly, the relative abundance of Ascomycota in high-salinity soil (*p* = 0.0017) increased compared with that in the low-salinity soil in this study (**Figure 10c**). This may be due to the fact that salt-tolerant bacteria such as Ascomycota can resist Na^+^ stress through special mechanisms (Egidi et al., 2019), that is, they can use ion pumps (such as H^+−^ATPASE and Na^+^/H^+^ retrotransporters) and ion channels to regulate the ion concentration difference inside and outside the cell, and excrete Na^+^ out of the cell, to adapt to the high-salinity environment. In summary, under stress conditions, soil microbial communities adapt to the environment by adjusting the community structure, while straw and biochar incorporation can enhance the stability of soil ecosystems to help to resist the stresses.

It has been found that biochar incorporation leads to a wider and higher intensity in the absorption peak of polysaccharides (Wang et al., 2024), and increases the accumulation of chlorophyll and photosynthetic metabolites (such as soluble sugar, sucrose, fructose, and starch) in crop leaves, enhancing the photosynthetic capacity of crops (Zahra et al., 2022). Zhang et al. (2023) showed that biochar incorporation increased the net photosynthetic rate of crops by improving stomatal restriction and intercellular carbon dioxide concentration in crop leaves compared with the control. Wang et al. (2021) showed that exogenous carbon addition significantly improved the structure, growth, and photosynthetic capacity of crop canopy, i.e., leaf number, leaf area expansion, leaf area duration, and canopy openness, which further leads to an increase in photosynthetic rate, leaf area index, chlorophyll content, and other photosynthetic traits of crops. Therefore, straw and biochar can enhance the photosynthetic capacity of crops, but how exogenous carbon incorporation improve soil physical, chemical, and biological properties and the accumulation of photosynthetic metabolites in crops needs to be further explored. In the follow-up research, we will further explore the effects of straw and biochar incorporation on the photosynthetic processes of cotton.

## 5 Conclusion

Straw and biochar incorporation, especially 4.5 t·hm^–2^ of biochar incorporation (B2), significantly reduced the salt content of soils with different salinization degrees, and increased the proportion of macroaggregates, compared with the control. Straw and biochar incorporation, especially 12 t·hm^−2^ of straw incorporation (C2), significantly increased the relative abundance of dominant bacteria such as Actinomycetes and Acidobacteria, and the diversity of soil microorganisms, compared with the control. Besides, straw and biochar incorporation significantly alleviated the salt stress by regulating the antioxidant defense system of cotton leaves (increasing the activities of SOD and CAT as well as decreasing the MDA content and POD activity). In conclusion, straw and biochar incorporation could significantly reduce the salt content of salinized soils by improving soil structure and regulating soil microbial community structure and abundance. Therefore, straw and biochar incorporation are effective strategies to remediate salinized soils, alleviate the salt stress in cotton, and achieve the efficient utilization of salinized soils by cotton planting.

Apart from biochar, modified biochar also shows good performance in the remediation of salinized soils. Therefore, more types of exogenous carbon will be combined to achieve better performance in the future.

## Data Availability

The data analyzed in this study is subject to the following licenses/restrictions: Confidentiality for a certain period of time. Requests to access these datasets should be directed to WL, 1048481036@qq.com.
